# Stigma, depression and pillbox return among adults living with HIV in rural Tanzania: A prospective cohort study

**DOI:** 10.1111/hiv.70090

**Published:** 2025-08-05

**Authors:** Robert Ndege, James Okuma, Bernard Kivuma, Raphael Magnolini, Leila Samson, Ezekiel Luoga, Elizabeth Senkoro, Olivia Kitau, Fiona Vanobberghen, Daniel H. Paris, Maja Weisser, the KIULARCO Study Group

**Affiliations:** ^1^ Biomedical Research and Clinical Trials Department Ifakara Health Institute Ifakara Tanzania; ^2^ Department of Medicine Swiss Tropical and Public Health Institute Allschwil Switzerland; ^3^ University of Basel Basel Switzerland; ^4^ St. Francis Regional Referral Hospital Ifakara Tanzania; ^5^ Arud Centre for Addiction Medicine Zurich Switzerland; ^6^ Institute of Primary Care, University Hospital Zurich University of Zurich Zurich Switzerland; ^7^ ViiV Healthcare GmbH Zug Switzerland; ^8^ Kilimanjaro Christian Medical Center Moshi Tanzania; ^9^ Breakthrough Cancer Research, Charities Institute Ireland Dublin Ireland; ^10^ Division of Infectious Diseases University Hospital Basel Basel Switzerland

**Keywords:** stigma depression, internalized stigma, perceived stigma, pillbox

## Abstract

**Background:**

Stigma remains a major barrier to accessing HIV care. We analysed the association between stigma and failed pillbox return, and described factors associated with stigma among adults living with HIV in rural Tanzania.

**Methods:**

From the Kilombero and Ulanga Antiretroviral Cohort we included newly diagnosed adults ≥15 years enrolled between June 2019 and September 2022 with a stigma assessment at 6–12 months and a clinical visit within 7 days. We assessed stigma using the adapted 7‐item Berger questionnaire. Logistic and linear regression models were used to identify factors associated with stigma and to determine the association between stigma and depression with failed pillbox return.

**Results:**

Among 241 participants (median age 37 years (IQR, 30–44) at enrolment), most were female (*n* = 170, 71%), WHO stage I (*n* = 132, 55%), had no pillbox at the clinical visit (*n* = 188, 78%), and remained in care at 2 years (*n* = 167, 69%). Participants with no pillbox had slightly higher stigma levels (9, IQR, 5–14) versus those who did (7, IQR, 4–13); this difference was not observed after adjustment (adjusted Odds Ratio 1.02 [95% CI 0.97, 1.07]; *p* = 0.38). Higher stigma was associated with higher depression (95% CI 0.48, 1.33, *p* < 0.01), HIV non‐disclosure (95% CI 2.58, 8.33, *p* < 0.01) and higher education (*p* < 0.01). Higher Body Mass Index (*p* < 0.01) and advanced WHO stage (*p* < 0.01) were associated with lower stigma.

**Conclusion:**

Failed pillbox return was not associated with stigma. However, stigma was associated with depression, HIV non‐disclosure and higher education. Stigma‐reduction interventions focused on mental health and disclosure are urgently needed in rural areas.

## BACKGROUND

While antiretroviral therapy (ART) remains highly effective, achieving viral suppression in 93% of people living with HIV on treatment in 2023, an estimated 23% of people living with HIV globally were not on ART in the same year [1]. Stigma, discrimination, gender‐based violence and social inequalities have been identified among factors preventing access to care [2]. Progress towards addressing the aforementioned barriers through the UNAIDS 10‐10‐10 targets—less than 10% of countries with restrictive laws and policies impeding access to HIV services, less than 10% of people living with HIV and key populations experiencing discrimination and stigma, and less than 10% experiencing gender inequality and violence—has been suboptimal. Nearly half (47%) of individuals from 42 countries with survey data collected between 2019 and 2023 reported discriminatory attitudes toward people living with HIV [1], and in 2023, people living with HIV across 25 countries reported experiencing stigma when seeking non‐HIV‐related services [3]. Moreover, a recent systematic review and meta‐analysis reported a pooled prevalence of 41% and 36% of HIV‐related perceived and internalized stigma in Africa [4]. The 2022–2023 Tanzania HIV Impact Survey reported 25.8% of adults and adolescents above the age of 15 to harbour discriminatory behaviour towards people living with HIV—with discrimination being more prevalent in rural (29.4%) compared with urban areas (20.1%) [5].

HIV‐related stigma [6] manifests in four main forms, including enacted stigma (direct experiences of discriminative acts), perceived stigma (belief that stigmatization against people living with HIV exists in the community), anticipated stigma (fear and/or expectation of discrimination upon disclosure of ones' HIV status) and internalized stigma (feelings of reduced self‐worth and shame in relation to HIV) [7]. All four have been associated with reduced access to HIV services (HIV testing, linkage to care, prevention of mother‐to‐child transmission of HIV services [8] and transition of children living with HIV to the adult clinic [9]), reduced ART adherence, poor viral control and retention in care [7, 9, 10, 11]. In addition, HIV‐related stigma has been associated with being female, tuberculosis co‐infection, living in rural areas, depression and non‐disclosure of HIV [12, 13, 14, 15].

Various tools for measuring HIV‐related stigma have been described [16, 17, 18, 19, 20], with the 40‐item Berger's stigma scale [16] being the most commonly used. It measures the four forms of stigma on a 4‐point Likert scale, with shorter (32‐, 25‐ and 12‐ item) versions being available [17, 21, 22]. However, integrating its delivery into routine HIV care remains challenging, particularly in low‐resource, high‐HIV burden settings, despite the availability of several promising stigma‐reduction interventions [23, 24].

Carrying the pillbox to the next clinical visit, which is required by most HIV programmes in sub‐Saharan Africa to measure adherence, has been associated with fear of accidental HIV status disclosure [25]—the rattling sounds of pills inside the container being perceived as a telltale sign of carrying ART [26]. In a previous study, we demonstrated an association between not returning the pillbox and lost to follow‐up (LTFU) [27]. Building on this, we aimed to examine the relationship between stigma and pillbox return, hypothesizing that people living with HIV experiencing HIV‐related stigma would be less likely to bring their pillbox to their clinical visit, possibly serving as an early marker to detect stigma. Additionally, we assessed the relationship between stigma and LTFU, depression and LTFU and described factors associated with HIV‐related stigma.

## METHODS

### Study design and population

We retrospectively analysed data from participants enrolled in the prospective Kilombero and Ulanga Antiretroviral Cohort (KIULARCO). KIULARCO is a rural HIV cohort established in 2005 at the St. Francis Regional Referral Hospital (SFRRH) HIV care and treatment centre, with over 13 000 people living with HIV ever enrolled, and approximately 3800 under active follow‐up in 2024 [28, 29]. Care and treatment of enrolled people living with HIV abide by the National HIV treatment guidelines [30]. Participants are followed up monthly until 6 months, and once stable, are seen twice yearly by a clinician. Participants are dispensed pillboxes containing pills until the next clinical visit, which, as per national guidelines, are required to be presented at every visit for pill counting and confirmation of the type of antiretroviral drugs prescribed. Laboratory monitoring, including cluster of differentiation‐4 (CD4) testing, is done at baseline. Yearly viral load measurements in the follow‐up period have been established since 2017. Demographic, clinical and laboratory information is documented in an electronic database (openMRS) as previously described [28, 29].

From February 2020, stigma‐directed services were introduced in KIULARCO for all individuals newly diagnosed with HIV aged 15 years and above, aiming at improving linkage to care and treatment outcomes [31]. Specifically, perceived and internalized stigma were addressed through the showing of an educational video and counselling sessions led by trained lay‐counsellors, namely, lay persons who openly live with HIV, trained by professional counsellors and medical personnel using national guideline‐aligned standard operating procedures [31]. In addition, group support therapy was offered, and automated appointment short message service (SMS) reminders were implemented. Stigma was assessed by the lay counsellors using a 7‐item Berger stigma scale, adapted and validated for the Tanzanian context [32]. It was measured on a 5‐point Likert‐type scale at 2–6 weeks and at 6–12 months after enrolment into KIULARCO [31]. Additionally, participants received depression assessments using the PHQ‐9 questionnaire [33] at the same time points.

### Study population

For this study, we included newly diagnosed adults aged 15 years and above, enrolled into KIULARCO and initiated on ART from June 2019 to September 2022, with a completed stigma assessment 6–12 months post‐ enrolment and a clinical visit within 7 days of the stigma assessment to capture pillbox return information. We excluded all individuals younger than 15 years and those without informed consent.

### Study objectives

The primary objective was to determine the association between stigma and pillbox return within the first year of enrolment into HIV care. Secondary objectives included identifying factors associated with stigma during the first year of care, and determining the association between stigma and LTFU within the first 2 years of enrolment. Additionally, we evaluated the association between depression and both pillbox return and loss to follow‐up within the same two‐year period.

### Definitions and covariates

Baseline was defined as the date of enrolment into KIULARCO. Pill counting was done at every clinical follow‐up visit. Failure to present the pillbox at a clinical follow‐up visit was defined as failed pillbox return. Results of the perceived and internalized stigma measurement with the adapted 7‐item Berger Stigma Scale were reported as scores ranging from 2 to 35 (lower scores indicating higher levels of stigma). Results of depression symptoms using the PHQ‐9 questionnaire were reported as a score ranging from 0 to 27, with higher PHQ‐9 scores indicating higher levels of depression.

LTFU was defined as the first episode of not attending a scheduled visit for more than 60 days after the appointment date within the first 2 years of enrolment into care, regardless of whether the participant re‐engaged with care afterwards.

Baseline covariates included age, gender, marital status, HIV status disclosure, HIV status of partner, education level, estimated distance from the clinic, Body Mass Index (BMI), WHO clinical stage, CD4 cell count and tuberculosis status. A tuberculosis diagnosis was defined as microbiological confirmation of *Mycobacterium tuberculosis* from any sample, or the initiation of anti‐tuberculosis medication in the presence of either a documented International Classification of Diseases‐10 (ICD‐10) code for tuberculosis or clinical signs suggestive of tuberculosis. Lack of an ICD‐10 code for tuberculosis with no prescription of anti‐tuberculosis medication was defined as no tuberculosis. If none of the above criteria were met, indeterminate tuberculosis was stated and treated as missing data. A window period of 1 month after enrolment was allowed for CD4 cell count, WHO stage and BMI measurements, and up to 3 months was allowed for tuberculosis diagnosis.

### Statistical methods

Baseline characteristics were summarized using median and interquartile ranges (IQRs) for continuous variables, and proportions and frequencies for categorical variables. Univariable and multivariable logistic regression models were used to determine the association between stigma and depression with pillbox return, stigma and LTFU, and depression and LTFU, with stigma and LTFU as the primary outcome. All baseline covariates—including depression—were included in the final multivariable logistic regression models. Participants with missing data were excluded from the respective analyses.

Univariable and multivariable linear regression models were used to identify factors associated with stigma, with beta coefficients (*β*) representing the expected change in stigma score for a one‐unit increase in the independent variable. Final models included depression and all baseline covariates. Robust standard errors were applied to account for heteroscedasticity, particularly due to small sample sizes in some categorical groups.

As stigma scores—including perceived and internalized stigma—were coded such that higher values reflected lower stigma, we reverse coded the scores by subtracting each score from one unit above the maximum possible score (e.g., 26—actual score for a 0–25 scale) for easier interpretation; therefore, higher values reflect higher stigma. Since stigma was included as a continuous variable without distinguishing between perceived and internalized stigma, as a sensitivity analysis, multivariable logistic regression models (adjusted for depression and all baseline covariates) were used to separately assess the associations between pillbox return and perceived stigma, and pillbox return and internalized stigma. To assess the internal consistency of the adapted Berger Stigma Scale within our study population, we computed Cronbach's alpha coefficients for the overall stigma scale and for perceived and internalized stigma subscales. All analyses were conducted using Stata version 18 (StataCorp. 2023. Stata Statistical Software: Release 18. College Station, TX: StataCorp LLC).

### Ethical considerations

The KIULARCO has an annually renewed ethical approval from the local Ifakara Health Institute Review Board (IHI/IRB/No16‐2006) and the National Institute for Medical Research (NIMR/HQ/R.8a/Vol.IX/620). Informed consent is routinely obtained from all participants prior to enrolment.

## RESULTS

**FIGURE 1:**
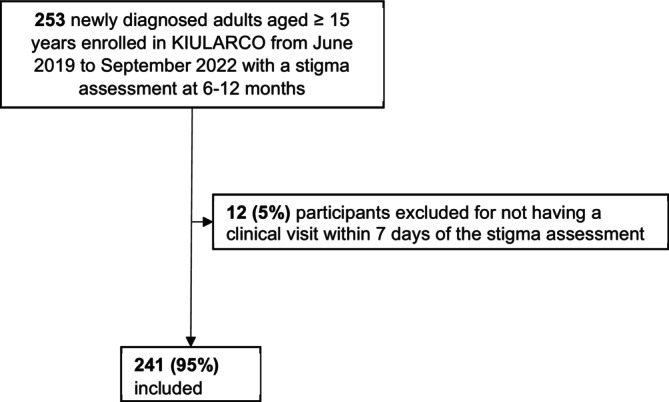
Patient flow chart. Stigma services implementation included: (i) Post‐diagnosis lay counselling services with testimonial videos of people living with HIV who were established in care (ii) Group support therapy and health education (iii) Automated appointment SMS reminders were sent to all participants with access to a mobile phone. KULARCO, Kilombero and Ulanga Antiretroviral Cohort.

Between June 2019 and September 2022, 253 adults living with HIV aged 15 years and above were enrolled into KIULARCO and received a stigma assessment at 6–12 months. We excluded 12 (5%) participants (Table S1) for not having a clinical visit within 7 days of the stigma assessment, resulting in 241 (95%) participants included in the study (Figure 1). At baseline, the median age of participants was 37 years (IQR 30, 44), the majority were female (170 [71%]), married (154 [64%]), in HIV WHO stage I (132 [55%]), lived ≤1 km from the clinic (150 [63%]), had disclosed their HIV status (166 [69%]), had a primary school level of education (200 [83%]) and did not bring back their pillbox to the scheduled clinical visit (188 [78%]). A total of 74 (31%) participants were lost to LTFU within 2 years of enrolment into care (Table 1). Study participants were generally similar to adults aged 15 years and above who were enrolled in the overall KIULARCO cohort and initiated on ART during the same time period (June 2019–September 2022) with respect to demographic and clinical characteristics. However, the study participants were more likely to have a lower WHO clinical stage and a primary or higher level of education. Additionally, we provide information on the 12 excluded participants (Table S1).

**TABLE 1:** Patients' characteristics by pillbox return among newly diagnosed adults living with HIV enrolled in KIULARCO.

Characteristics	Total	Pillbox return^a^	
		No	Yes
	*N* = 241	*N* = 188 (78%)	*N* = 53 (22%)
Age, years, median (IQR)	37 (30, 44)	36 (30, 43)	38 (31, 47)
Sex, female, *n* (%)	170 (71%)	130 (69%)	40 (75%)
Marital status, *n* (%)			
Married/cohabiting	154 (64%)	124 (66%)	30 (57%)
Never married	16 (7%)	12 (6%)	4 (8%)
Separated/divorced/Widowed	63 (26%)	46 (25%)	17 (32%)
Other	8 (3%)	6 (3%)	2 (4%)
Disclosed HIV status, *n* (%)	166 (69%)	133 (71%)	33 (62%)
Disclosed to,^b^ *n* (%)			
Partner	85 (35%)	68 (36%)	17 (32%)
Friend	5 (2%)	5 (3%)	0 (0%)
Relative	82 (34%)	63 (34%)	19 (36%)
Support group	1 (0%)	1 (0%)	0 (0%)
Other person	8 (3%)	6 (3%)	2 (4%)
Partner status, *n* (%)			
Positive	45 (19%)	35 (19%)	10 (19%)
Negative	32 (13%)	25 (13%)	7 (13%)
Not tested	59 (24%)	42 (22%)	17 (32%)
Unknown	16 (7%)	12 (6%)	4 (8%)
Not applicable	89 (37%)	74 (39%)	15 (28%)
Education, *n* (%)			
None	15 (6%)	12 (6%)	3 (6%)
Primary school	200 (83%)	157 (84%)	43 (81%)
Secondary school or higher	26 (11%)	19 (10%)	7 (13%)
Distance from the clinic,^c^ *n* (%)			
≤1 km	150 (63%)	117 (63%)	33 (64%)
2–<50 km	65 (27%)	56 (30%)	9 (17%)
≥50 km	23 (10%)	13 (7%)	10 (19%)
Body mass index (BMI),^d^ kg/m^2^, median (IQR)	23 (20, 27)	23 (20, 27)	22 (20, 25)
WHO clinical stage, *n* (%)			
I	132 (55%)	111 (59%)	21 (40%)
II	57 (24%)	41 (22%)	16 (30%)
III	40 (17%)	29 (15%)	11 (21%)
IV	12 (5%)	7 (4%)	5 (9%)
Tuberculosis (yes), *n* (%)	19 (8)	11 (6%)	8 (15%)
CD4 count,^e^ cells/mm^3^, median (IQR)	264 (134, 446)	263 (138, 446)	270 (130, 424)
Depression score (range 0–27), median (IQR)	1 (0, 3)	1 (0, 3)	1 (0, 4)
Any Stigma score (range 2–35), median (IQR)	9 (5–13)	9 (5–14)	7 (4–13)
Internalized Stigma score (range 2–10)	3 (1–5)	3 (1–5)	4 (1–5)
Perceived Stigma score (range 0–25)	6 (3–10)	6 (3–10)	5 (2–11)
Status at 2 years^f^			
Lost to follow up (LTFU)	74 (31%)	57 (30%)	17 (32%)
Active in care	167 (69%)	131 (70%)	36 (68)

*Note*: Results are numbers and column % of those with non‐missing data. A window period of 1 month after enrolment was allowed for CD4 cell count, WHO stage and BMI measurements, and up to 3 months was allowed for tuberculosis diagnosis.

Abbreviations: IQR, Interquartile Range; WHO stage, World Health Organization HIV clinical staging.

^a^ Pillbox return was defined as the act of carrying and presenting the pillbox in the next scheduled clinical follow‐up visit.

^b^ A participant can disclose to more than one person.

^c^ 3 participants did not have distance from clinic information.

^d^ 2 participants did not have baseline BMI information.

^e^ 19 participants did not have baseline CD4 information.

^f^ Transfer out was termed active in care; 2 participants transferred out during the 2‐year period.

### Predictors of stigma

In the multivariable model, one‐unit increase in depression was independently associated with a 0.91 point increase in level of stigma (95% CI 0.48, 1.33; *p* < 0.01). Similarly, those who had not disclosed their HIV status had 5.45 points higher stigma levels than those who had disclosed (95% CI 2.58, 8.33; *p* < 0.01). Participants with a primary or secondary/higher level of education had 6.16 points (95% CI 3.35, 8.97), and 5.57 points (95% CI 1.15, 9.99) higher stigma levels, respectively, compared with those with no formal education (*p* < 0.01).

**FIGURE 2:**
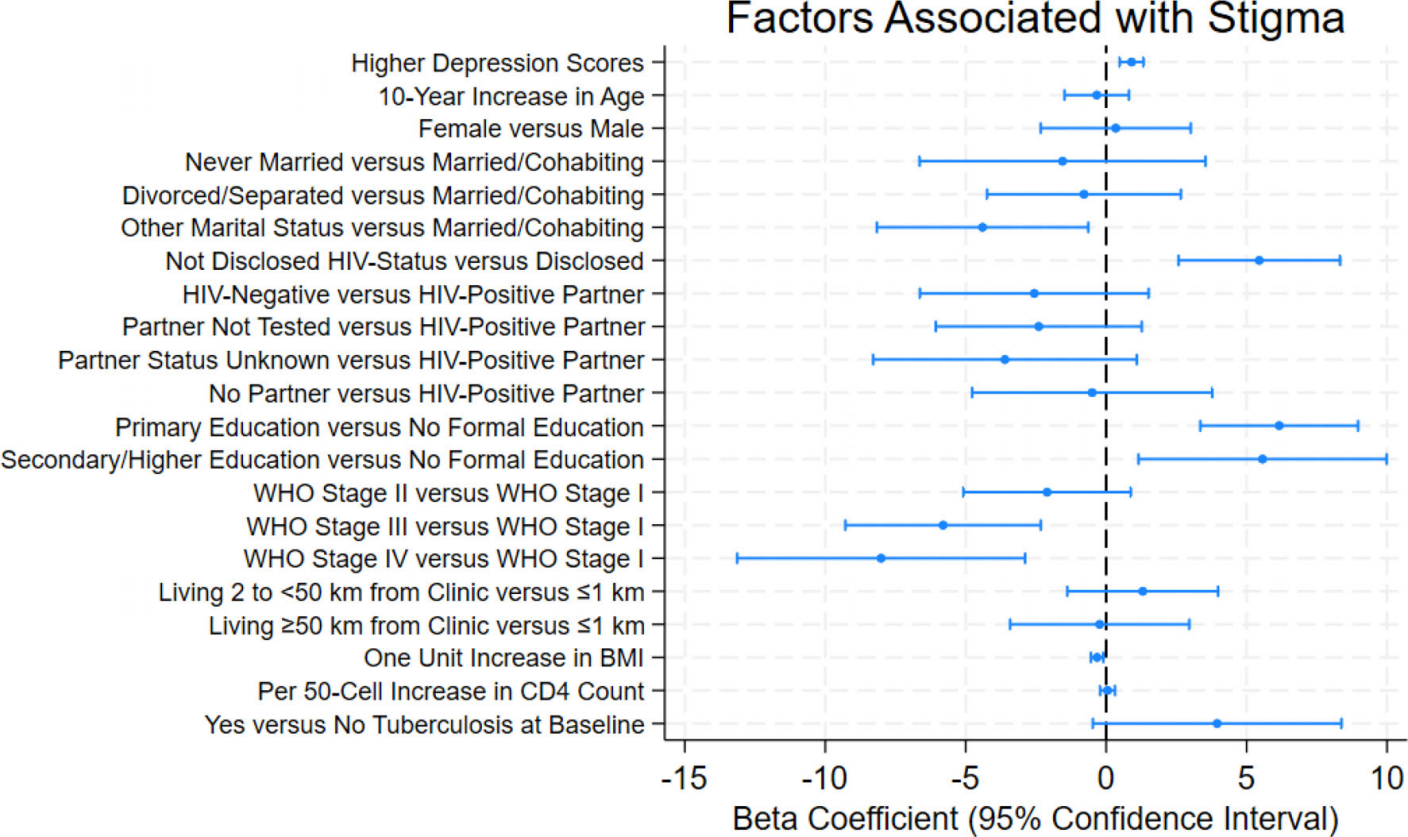
Forest Plot of factors associated with stigma among newly diagnosed adults living with HIV enrolled in KIULARCO. BMI, body mass index; CD4, cluster of differentiation 4; Km, kilometres; WHO, World Health Organization.

By contrast, each one‐unit higher BMI was associated with a 0.32 point lower stigma score (95% CI −0.54, −0.10; *p* < 0.01). Participants with advanced WHO clinical stages of stage II, III or IV had 2.10 points (95% CI −5.08, 0.88), 5.81 points (95% CI −9.29, −2.32) and 8.01 points (95% CI −13.14, −2.89) lower stigma levels, respectively, compared with participants with WHO clinical stage I (*p* < 0.01) (Table 2 and Figure 2).

**TABLE 2:** Factors associated with stigma among newly diagnosed adults living with HIV enrolled in KIULARCO.

Baseline Covariates	Univariable^a^ coefficient (95% CI), *p*‐value (*n* = 241)	Multivariable coefficient (95% CI), *p*‐value (*n* = 216^b^)
Depression	0.70 (0.30, 1.10), <0.01	0.91 (0.48, 1.33), <0.01
Age^c^	−0.68 (−1.69, 0.34), 0.19	−0.33 (−1.48, 0.81), 0.57
Sex, female	0.55 (−1.96, 3.06), 0.67	0.34 (−2.33, 3.01), 0.80
Marital status (overall)	0.33	0.15
Married/cohabiting	Ref	Ref
Never married	1.17 (−3.48, 5.82)	−1.55 (−6.64, 3.54)
Separated/divorced/widowed	−0.86 (−3.51, 1.79)	−0.79 (−4.24, 2.66)
Other	−5.45 (−11.88, 0.97)	−4.40 (−8.17, −0.64)
Disclosure, no	2.99 (0.54, 5.43), 0.02	5.45 (2.58, 8.33), <0.01
Partner status	0.87	0.39
Positive	Ref	Ref
Negative	−1.19 (−5.31, 2.94)	−2.56 (−6.63, 1.52)
Not tested	−0.56 (−4.09, 2.97)	−2.40 (−6.07, 1.27)
Unknown	−2.81 (−8.00, 2.38)	−3.60 (−8.29, 1.09)
Not applicable	−0.73 (−3.99, 2.53)	−0.50 (−4.77, 3.78)
Education^d^	0.05	<0.01
None	Ref	Ref
Primary school	5.92 (1.21, 10.62)	6.16 (3.35, 8.97)
Secondary school or higher	5.61 (−0.09, 11.31)	5.57 (1.15, 9.99)
Distance from the clinic	0.52	0.58
≤1 km	Ref	Ref
2–<50 km	1.36 (−1.29, 4.01)	1.30 (−1.38, 3.99)
≥50 km	−0.66 (−4.65, 3.33)	−0.23 (−3.42, 2.96)
Body mass index	−0.04 (−0.26, 0.18), 0.72	−0.32 (−0.54, −0.10), <0.01
WHO stage at baseline	0.15	<0.01
I	Ref	Ref
II	−1.53 (−4.32, 1.27)	−2.10 (−5.08, 0.88)
III	−2.97 (−6.15, 0.22)	−5.81 (−9.29, −2.32)
IV	−4.32 (−9.64, 1.00)	−8.01 (−13.14, −2.89)
CD4 count^e^	0.21 (−0.02, 0.45)	0.05 (−0.21, 0.31)
Tuberculosis status at baseline	0.01 (−4.25, 4.26)	4.03 (−0.50, 8.57)

^a^ Linear regression was used to determine the factors that are associated with stigma. Stigma scores were inverted (multiplied by −1) for interpretability; higher values reflect greater stigma.

^b^ *n* = 216 for multivariable models due to missing covariates (3 missing distance, 2 BMI, 19 CD4, 2 TB status).

^c^ Age scaled per 10 years increase.

^d^ Heteroskedasticity was present; robust standard errors were used.

^e^ CD4 scaled per 50 cells/mm 3 increase.

### Stigma and depression association with failed pillbox return

Participants who did not bring back the pillbox had a higher median stigma score (9 [IQR 5, 14]) compared with those who brought back the pillbox (7 [IQR 4, 13]), but with overlapping IQR (Table 1). There was no evidence of an association between pillbox return and stigma (aOR, 1.02 [95% CI 0.97, 1.07]; *p* = 0.38). The results remained unchanged after independently assessing for internalized (aOR 0.90 [95% CI 0.77, 1.04], *p* = 0.16) and perceived stigma (aOR 1.02 [95% CI 0.97, 1.07], *p* = 0.40) in separate regression models (Table 3). There was no difference in levels of depression between participants based on pillbox return.

**TABLE 3:** Stigma and depression association with failed pillbox return among newly diagnosed adults living with HIV enrolled in KIULARCO.

Covariates	Univariable, OR (95% CI)^a^, *p*‐value (*n* = 241)	Multivariable, aOR (95% CI),^a^, ^b^ *p*‐value (*n* = 216)
Any stigma	1.01 (0.98, 1.05), 0.57	1.02 (0.97, 1.07), 0.38
Internalized stigma^c^	0.94 (0.84, 1.07), 0.36	0.90 (0.77, 1.04), 0.16
Perceived stigma^d^	1.02 (0.98, 1.06), 0.38	1.02 (0.97, 1.07), 0.40
Depression	0.95 (0.85, 1.05), 0.28	0.92 (0.80, 1.06), 0.27

^a^ Odds ratios (OR), 95% confidence intervals (CI) and *p*‐values obtained from logistic regression.

^b^ Adjusted for baseline covariates including age, sex, marital status, disclosure status, partner status, education, distance from the clinic, Body Mass Index (BMI), WHO stage, CD4 count, tuberculosis (TB) status, and depression. *n* = 216 due to missing covariates (3 missing distance, 2 BMI, 19 CD4, 2 TB status).

^c^ Internalized stigma is defined as feelings of reduced self‐worth and shame in relation to HIV.

^d^ Perceived stigma is defined as the belief that stigmatization against people living with HIV exists in the community.

### Association between stigma or depression and lost to follow up

We did not find any evidence of an association between stigma or depression with LTFU (stigma: aOR 1.00 [95% CI 0.97, 1.04], *p* = 0.87; depression: aOR 1.06 [95% CI 0.94, 1.20], *p* = 0.33) (Table 4).

**TABLE 4:** Association between stigma and depression with loss to follow‐up among newly diagnosed adults living with HIV enrolled in KIULARCO.

Baseline covariates	Stigma	Depression
	Multivariable model, aOR (95% CI)^a^, *p*‐value (*n* = 216)	Multivariable model, aOR (95% CI)^a^, *p*‐value (*n* = 216)
Stigma/depression	1.00 (0.97, 1.04), 0.87	1.06 (0.94, 1.20), 0.33
Age^b^	1.04 (1.01, 1.07), 0.02	1.04 (1.01, 1.07), 0.02
Sex, female	1.40 (0.66, 2.97), 0.38	1.34 (0.62, 2.86), 0.46
Marital status	0.03	0.02
Married/cohabiting	Ref	Ref
Never married	2.73 (0.74, 10.05)	2.65 (0.71, 9.90)
Separated/divorced/widowed	2.01 (0.85, 4.77)	2.05 (0.87, 4.85)
Other	11.61 (2.00, 67.32)	11.74 (2.07, 66.53)
Disclosure, no	1.02 (0.47, 2.22), 0.97	1.02 (0.48, 2.17), 0.96
Partner status	0.67	0.68
Positive	Ref	Ref
Negative	0.84 (0.26, 2.70)	0.82 (0.25, 2.64)
Not tested	0.94 (0.34, 2.56)	0.91 (0.33, 2.48)
Unknown	0.85 (0.18, 4.10)	0.91 (0.19, 4.35)
No partner	0.50 (0.17, 1.48)	0.50 (0.17, 1.48)
Education	0.89	0.91
None	Ref	Ref
Primary school	0.89 (0.23, 3.39)	0.94 (0.25, 3.52)
Secondary school or higher	1.13 (0.22, 5.75)	1.19 (0.23, 6.01)
Distance from the clinic	0.08	0.07
≤1 km	Ref	Ref
2–<50 km	2.08 (1.03, 4.20)	2.16 (1.06, 4.39)
≥50 km	0.78 (0.26, 2.34)	0.79 (0.26, 2.40)
Body mass index	1.00 (0.93, 1.07), 0.97	1.00 (0.93, 1.07), 0.99
WHO stage	0.56	0.64
I	Ref	Ref
II	1.70 (0.75, 3.85)	1.58 (0.69, 3.63)
III	1.01 (0.34, 3.02)	0.93 (0.31, 2.75)
IV	1.71 (0.32, 9.08)	1.56 (0.30, 8.23)
CD4 cell count^c^	1.00 (1.00, 1.00), 0.81	1.00 (1.00, 1.00), 0.89
Tuberculosis	1.48 (0.41, 5.35), 0.55	1.53 (0.42, 5.50), 0.52

*Note*: A window period of 1 month after enrolment was allowed for CD4 cell count, WHO stage and BMI measurements, and up to 3 months was allowed for tuberculosis diagnosis.

^a^ Adjusted Odds Ratios (aOR), 95% Confidence Intervals (CI) and *p* values obtained from logistic regression. Adjusted for other baseline covariates shown; *n* = 216 due to missing covariates (3 missing distance, 2 BMI, 19 CD4 2 TB status).

^b^ Age scaled 10 years increase.

^c^ CD4 scaled 50 cells/mm 3 increase.

### Internal consistency of the adapted Berger Stigma Scale

The overall 7‐item Berger Stigma Scale demonstrated low internal consistency, with a Cronbach's alpha coefficient of 0.58. The internal consistency for the 2‐item internalized stigma subscale was similarly low, with an alpha of 0.58. The 5‐item perceived stigma subscale showed the highest internal consistency, although it remained modest, with a Cronbach's alpha of 0.60.

## DISCUSSION

We report on the first study to assess the association between pillbox return and stigma. We found a trend towards lower stigma levels among participants who brought back the pillbox to the next scheduled visit; however, after adjustment for confounders, no evidence of an association between pillbox return and stigma remained. Higher stigma was associated with depression, HIV non‐disclosure and higher education, while higher BMI and advanced WHO stage were associated with lower stigma. There were no differences in depression levels between the two groups.

We could not ascertain our hypothesis of a possible association between pillbox return and stigma. With 78% of participants not bringing back the pillbox and an overall low level of stigma, our ability to detect a significant difference was limited. In addition, stigma was not associated with LTFU, possibly due to the overall small proportion of participants that were LTFU, which might be different from our previous study on the association of pillbox return with LTFU [27]. Additionally, the stigma interventions that were implemented in the clinic [31] during this period might have impacted our findings, which are in line with a study from Kwazulu‐Natal, South Africa, in which the authors could also not find an association between self‐reported stigma and LTFU among adults living with HIV [34]. The authors attributed their findings to reduced levels of stigma in a community with a high HIV burden. Contrarily, a recent literature review on the impact of stigma on engagement and retention in HIV care among children, adolescents and young adults found stigma to negatively affect retention in care within this population [9].

Depression was positively associated with stigma in our study, which is in line with studies conducted in similar settings [35, 36] and two large meta‐analyses [12, 37]. A systematic review of studies from South Africa found mixed results on the relation between stigma and depressive symptoms among people living with HIV, suggesting that the relationship could be bidirectional [38]. In our study, non‐disclosure of HIV status was positively associated with stigma in line with a recent systematic review [12]. While we found higher education levels to be associated with increased stigma, studies from South Africa have reported mixed results regarding this relationship [39, 40]. Additionally, a systematic review also reported higher education levels to be associated with lower HIV‐related stigma [12]. Our findings could be explained by the rural nature of our study setting with a low HIV prevalence of 2.5% [5]. A higher education level in this setting could be synonymous with a better understanding of HIV and an increased awareness or anticipation of stigma.

An increase in BMI was associated with reduced stigma. This finding aligns with existing literature on the perception of weight gain among people living with HIV. Weight gain is often seen as a sign of restored health, an indication of the effectiveness of ART, and as a protective factor against accidental disclosure of HIV status [41]. Additionally, in certain cultures, it is associated with attractiveness and a higher social standing [42]. These factors could explain why we found stigma to decrease with weight gain. Lastly, having an advanced HIV disease at baseline was also associated with a decrease in stigma. A higher WHO clinical stage could be comparable to living with HIV for a longer period of time, which has been associated with decreased levels of stigma [12].

The main strength of our study is its conduction under real world conditions with the use of routinely collected data. However, our study had limitations. Firstly, a large proportion of our study participants did not bring back the pillbox. From the pragmatic nature of our study, we believe this finding applies to similar settings. Moreover, since returning the pillbox was not required for service access, most participants may have chosen not to return it as a coping mechanism to mitigate fears of accidental or unwanted HIV status disclosure, as reported in previous studies [25, 26, 43]. However, this limited our ability to find a significant difference in stigma between the groups, and may undermine the usefulness of pillbox return as a stigma measure. Secondly, the Berger scale only assessed perceived and internalized stigma; we were therefore unable to analyse other forms of HIV‐related stigma, hence limiting the generalizability of our results. However, results from a national survey in South Africa among people living with HIV found perceived and internalized stigma to be the most common forms [44], and a second study conducted among young people living with HIV in Zambia did not find an association between enacted stigma and health outcomes [45]. Thirdly, the implementation of stigma services that were introduced in the clinic could have impacted LTFU rates, possibly affecting the association of stigma and LTFU. Lastly, the adapted Berger Stigma Scale demonstrated low internal consistency in this study, which may limit the reliability of the stigma measurements. This may be due to the small number of items, cultural differences from the original validation population, and/or administration by lay counsellors. These factors could have influenced how participants understood and responded, contributing to the limited reliability. Further validation and refinement of the scale are recommended for this context.

To conclude, perceived and internalized stigma were not associated with pillbox return. Depression, non‐disclosure of HIV status, and higher levels of education were associated with increased stigma levels. Interventions that promote HIV status disclosure and support mental health should be considered in stigma‐directed interventions in rural settings.

## AUTHOR CONTRIBUTIONS

RN and MW conceptualized the study. RN, JO and FV contributed to the data analysis. RN wrote the first draft of the manuscript. ES, OK, RM, BK and DHP contributed to the study design and reviewed the manuscript. All the authors read and approved the final version of the manuscript.

## CONFLICT OF INTEREST STATEMENT

Since March 2025, RM is employed by ViiV Healthcare Switzerland. This work was conducted before his start at ViiV. ViiV was not involved in the design of the study, the data collection, analysis, interpretation and manuscript writing, and had no access to the dataset. All other authors declare no competing interests.

## Supporting information


**Data S1.** Supporting information.

## Data Availability

The data that support the findings of this study are available from the corresponding author upon reasonable request.
