# Supplementary material for: Stigma, depression and pillbox return among adults living with HIV in rural Tanzania: A prospective cohort study
Source: HIV Med. 2025 Aug 5;26(10):1600–11. doi: 10.1111/hiv.70090 (PMC12497930; doi:10.1111/hiv.70090)
Supplement: Supplementary file 1 — Data S1. Supporting information. [file HIV-26-1600-s001.docx]

**Supplementary Table 1:** Comparison of baseline characteristics between study participants and participants enrolled into the KIULARCO Cohort during the study period (June 2019 to September 2022)

| Baseline Covariates | Participants enrolled into KIULARCO between June 2019 and September 2022 | Study Population | Excluded  Participants |
| --- | --- | --- | --- |
|  | N=1,234 | N=241 | N = 12 |
| Age, years | 38 (29-47) | 37 (30-44) | 45 (31-52) |
| Sex  Male  Female | 419 (34%)  815 (66%) | 71 (29%)  170 (71%) | 1 (8%)  11 (92%) |
| Marital status  Married/cohabiting  Never married  Separated/divorced  Other | 790 (64%)  95 (8%)  320 (26%)  29 (2%) | 154 (64%)  16 (7%)  63 (26%)  8 (3%) | 7 (58%)  2 (17%)  3 (25%)  0(0%) |
| Level of education  none/other  Primary School  Secondary or higher | 141 (11%)  946 (77%)  147 (12%) | 15 (6%)  200 (83%)  26 (11%) | 0 (0%)  10 (83%)  2 (17%) |
| Disclosure of HIV status  No  Yes | 371 (33%)  760 (67%) | 75 (31%)  166 (69%) | 2 (17%)  10 (83%) |
| Partner status  Positive  Negative  Not tested  Unknown  No Applicable | 195 (17%)  145 (13%)  199 (18%)  119 (11%)  473 (42%) | 45 (19%)  32 (13%)  59 (24%)  16 (7%)  89 (37%) | 4 (33%)  0 (0%)  2 (17%)  0 (0%)  6 (50%) |
| Distance from clinic* | 1 (1-30) | 1 (1-25) | 1 (1-1) |
| Body Mass Index, kg/m2 | 23 (20-27) | 23 (20-27) | 25 (21-27) |
| WHO Clinical Stage  I  II  III  IV | 594 (52%)  199 (17%)  185 (16%)  165 (14%) | 132 (55%)  57 (24%)  40 (17%)  12 (5%) | 7 (58%)  4 (33%)  0 (0%)  1 (8%) |
| Tuberculosis status at Baseline  No  Yes | 1,097 (91%)  104 (9%) | 220 (92%)  19 (8%) | 12 (100%)  0 (0%) |
| CD4 count, cells/mm3 | 276 (123-482) | 264 (134-446) | 281 (136-448) |

* 2 participants were missing distance information
